# Epidemiological characteristics and environmental surveillance of human psittacosis in Lishui City, Zhejiang Province, China (2021–2024)

**DOI:** 10.3389/fmicb.2026.1769696

**Published:** 2026-04-08

**Authors:** Xiuying Chen, Li Gong, Yuzhong Lu, Wujing Liu, Fuming Liu, Qi Li, Lingbo Wang, Liyang Qiu, Deyong Zhang, Xialiang Ye

**Affiliations:** 1 Lishui Center for Disease Control and Prevention, Lishui, Zhejiang, China; 2 Zhejiang Provincial Center for Disease Control and Prevention, Hangzhou, Zhejiang, China

**Keywords:** *Chlamydia psittaci*, environmental surveillance, epidemiology, pneumonia, *psittacosis*, zoonosis

## Abstract

**Introduction:**

Psittacosis, caused by *Chlamydia psittaci*, is an underdiagnosed zoonosis that can lead to severe pneumonia and fatal outcomes. In China, traditional poultry farming poses substantial risks for avian-to-human transmission, yet comprehensive epidemiological evidence is scarce. To address this gap, we aimed to define the local epidemiology, risk factors, and environmental reservoirs of human *psittacosis* in Lishui City, Zhejiang Province.

**Methods:**

We conducted a multi-source epidemiological study (2021–2024) integrating surveillance data, clinical records, contact investigations, and environmental sampling. Cases were confirmed by quantitative polymerase chain reaction (qPCR) or metagenomic next-generation sequencing (mNGS).

**Results:**

We identified 28 laboratory-confirmed cases, showing annual fluctuations in reported case numbers. Infections, mostly confirmed by mNGS, were predominantly sporadic among elderly agricultural workers (mean age 62.6 years), with 96.4% reporting recent poultry exposure. All patients presented with pneumonia; 64.3% developed severe disease, resulting in three deaths. The median diagnostic delay—from symptom onset to diagnosis—was 12 days. A household cluster of three cases was detected; however, no secondary transmission occurred among 205 close contacts outside the household. *C. psittaci* DNA was detected in 14.79% (21/142) of environmental samples, with the highest number of cases detected in duck manure samples, with the highest positive rate (26.7%). Phylogenetic analysis of 20 ompA gene sequences revealed a predominantly genotype A and the waterfowl-TW genotype, which are closely related to strains from southern China.

**Discussion:**

*Psittacosis* in Lishui presents as a sporadic but clinically severe disease in older rural residents. The high frequency of severe pneumonia and prolonged diagnostic delay underscores an urgent need to improve clinical suspicion and access to molecular diagnostics. Detection of *C. psittaci* nucleic acid in environmental samples suggests possible environmental contamination; however, viability and transmissibility were not assessed.

## Introduction

*Psittacosis* (ornithosis) is a zoonotic infectious disease caused by *Chlamydia psittaci*, an obligate intracellular Gram-negative bacterium (Beeckman and Vanrompay, 2009). The pathogen predominantly circulates among avian species and is transmitted to humans mainly through inhalation of aerosolized secretions, fecal matter, or feather dust (Wang et al., 2024). Although cases of person-to-person transmission are rare, they have also been reported (Zhang et al., 2022). Clinical presentation in humans varies substantially, ranging from mild influenza-like illness to severe pneumonia and systemic inflammatory complications. Without prompt diagnosis and appropriate treatment, these conditions can be fatal (Dembek et al., 2023).

In China, sporadic cases and outbreaks reported across both rural and urban settings underscore its emerging public health significance (Yang et al., 2024). Nevertheless, the actual burden of disease is likely underestimated, given that clinical features are nonspecific, awareness among clinicians is limited, and molecular diagnostic tools such as qPCR, and metagenomic next-generation sequencing are not routinely available in many healthcare facilities (Knittler and Sachse, 2015). Consequently, *psittacosis* is often omitted from the differential diagnosis of community-acquired pneumonia, resulting in delayed detection and inadequate patient management (Wen et al., 2025). Molecular epidemiological studies commonly employ sequencing of the outer membrane protein A (ompA) gene to characterize genetic diversity and host-associated genotypes of *C. psittaci* (Hou et al., 2024; Soon et al., 2025). Multiple genotypes have been identified, including genotype A, which is frequently associated with psittacine birds and human infections, as well as waterfowl-associated lineages such as the TW genotype (Mattmann et al., 2019).

Lishui City, a mountainous area in southwestern Zhejiang Province, presents a high-risk environment for zoonotic transmission due to common practices of backyard poultry farming, especially among elderly rural residents (Sassa-O'Brien et al., 2025). These factors favor spillover from avian to human hosts of *C. psittaci* (Veletzky et al., 2021). Despite the probable elevated risk, systematic epidemiological studies in this region are scarce, and integrated approaches combining clinical, epidemiological, and environmental assessments have not yet been conducted (Jin et al., 2023). Several recent surveillance reports from Europe and Asia have highlighted fluctuations in *psittacosis* notifications and emphasized the need for strengthened surveillance and molecular characterization of circulating *Chlamydia psittaci* strains (Herrmann et al., 2024; Raven et al., 2025). These findings underscore the importance of integrating epidemiological investigation with molecular typing to better understand transmission dynamics and potential zoonotic sources (Deng et al., 2024).

To bridge this gap, we are implementing a comprehensive, multi-source epidemiological investigation in Lishui from August 2021 to December 2024. By integrating case surveillance, clinical characterization, environmental sampling, and molecular testing, this study aims to identify major risk factors, particularly those related to domestic poultry exposure, and assess the extent of environmental contamination with *C. psittaci* in affected rural communities. Our findings are expected to provide critical insights into the local transmission dynamics of *psittacosis* and facilitate the development of evidence-based surveillance and preventive public health strategies.

## Materials and methods

### Study design and ethical approval

A retrospective, multi-source epidemiological study was conducted in Lishui City from January 2021 to December 2024. Routine statutory surveillance data were retrospectively retrieved from January 2021 onward, while the integrated multi-source field investigation (including structured exposure assessment and systematic environmental sampling) was formally initiated in August 2021. Data were integrated from statutory case surveillance, clinical record reviews, contact tracing, and environmental sampling, and molecular confirmation was employed for case validation. The study protocol received approval from the Lishui Municipal Health Commission Ethics Committee, and informed consent was obtained from all participants or their proxies.

### Case definition and ascertainment

Eligible cases were individuals reported to the national infectious disease surveillance system with laboratory-confirmed *Chlamydia psittaci* infection via nucleic acid-based methods-quantitative PCR (qPCR) or mNGS-following standard public health protocols. Standardized notification forms were completed by clinicians, and duplicate entries were resolved using unique identifiers.

### Data collection and variables

For each confirmed case, trained investigators collected data from medical records using a structured template. The variables encompassed demographic characteristics (age and sex), occupation, symptom onset date, dates of healthcare seeking (first consultation and diagnosis), hospitalization status, and clinical severity. Exposure histories, particularly those related to poultry ownership, rearing, slaughtering, or cage cleaning, were evaluated using a standardized epidemiological questionnaire, which was administered during interviews or via proxy when necessary. Time intervals—symptom onset to first consultation, first consultation to diagnosis, and symptom onset to diagnosis—were computed in days from the recorded dates.

### Contact tracing and follow-up

Close contacts were identified based on exposures in the household, caregiving settings, and other relevant environments. Each contact was placed under mandatory medical observation for 14 days from the last exposure date. Daily monitoring for fever and respiratory symptoms was conducted by healthcare personnel under municipal health guidelines. Symptomatic individuals were referred to designated clinics for laboratory testing.

### Environmental sampling and laboratory methods

Environmental and animal sampling was conducted as emergency, case-triggered convenience sampling rather than random or systematic surveillance. Sampling was initiated after laboratory confirmation of selected cases. Sites were selected based on patient-reported exposure history. Not all confirmed cases underwent environmental investigation. The number and type of specimens collected per site were determined according to poultry species, number of birds, and environmental conditions. Sampling was generally conducted within 3–7 days after case confirmation. Repeat sampling was not routinely performed. Environmental investigations were carried out in households of confirmed cases and other epidemiologically significant sites. Environmental investigations followed a risk-based targeted sampling strategy. Sampling was conducted in households of confirmed cases and epidemiologically linked sites rather than through systematic population-representative surveillance across all counties. The primary objective was source attribution and assessment of contamination in exposure settings. Sampling focused on avian species and their habitats, including domestic poultry, and peri-domestic environments. Specimens included nasopharyngeal and cloacal swabs from poultry; fresh fecal samples; drinking water; and surface samples from cages and equipment. All samples were collected aseptically, maintained at 2–8 °C, and transported with a documented chain of custody. Biosafety Level 2 (BSL-2) practices were adhered to during sample processing, following Lishui CDC protocols. *C. psittaci* was detected using species-specific quantitative PCR (qPCR) targeting the ompA gene and/or nested PCR amplification following validated laboratory protocols. The primer and probe sequences were designed based on previously published protocols (Hogerwerf et al., 2017; Pantchev et al., 2009) and are listed in Supplementary Table S1. A cycle threshold (Ct) value ≤38 was considered positive. Each qPCR run included positive controls, negative controls, and extraction blanks. To prevent cross-contamination, nucleic acid extraction, reagent preparation, and amplification were conducted in physically separated laboratory areas under Biosafety Level 2 (BSL-2) conditions. Repeat testing was performed for samples with borderline Ct values. mNGS was utilized in cases with sufficient nucleic acid yield or unresolved diagnoses. Each assay included positive and negative controls, and repeat testing was performed to clarify equivocal results.

### Metagenomic next-generation sequencing

For metagenomic next-generation sequencing (mNGS), libraries were prepared using the Illumina Nextera XT DNA Library Preparation Kit and sequenced on an Illumina NextSeq 550 platform (paired-end 150 bp) (Li et al., 2021). The workflow followed previously described clinical diagnostic protocols (Xiao et al., 2021; Yin et al., 2021). Approximately 15–25 million reads were generated per sample. Host-derived reads were removed by alignment to the human reference genome (hg38). Remaining reads were mapped against curated microbial reference databases using a validated bioinformatics pipeline. A pathogen was considered detected when the number of non-overlapping reads mapped to species-specific genomic regions exceeded predefined background thresholds. Specifically, detection required ≥3 non-overlapping reads uniquely aligned to the reference genome and a reads-per-million (RPM) value at least 10-fold higher than that observed in negative controls processed within the same sequencing batch. Background levels were estimated using extraction blanks and no-template controls included in each sequencing run. Microorganisms detected at similar levels in negative controls were considered potential contaminants and excluded from interpretation unless their read counts were substantially higher in clinical specimens.

### Cluster and phylogenetic analysis

**Figure 1:**
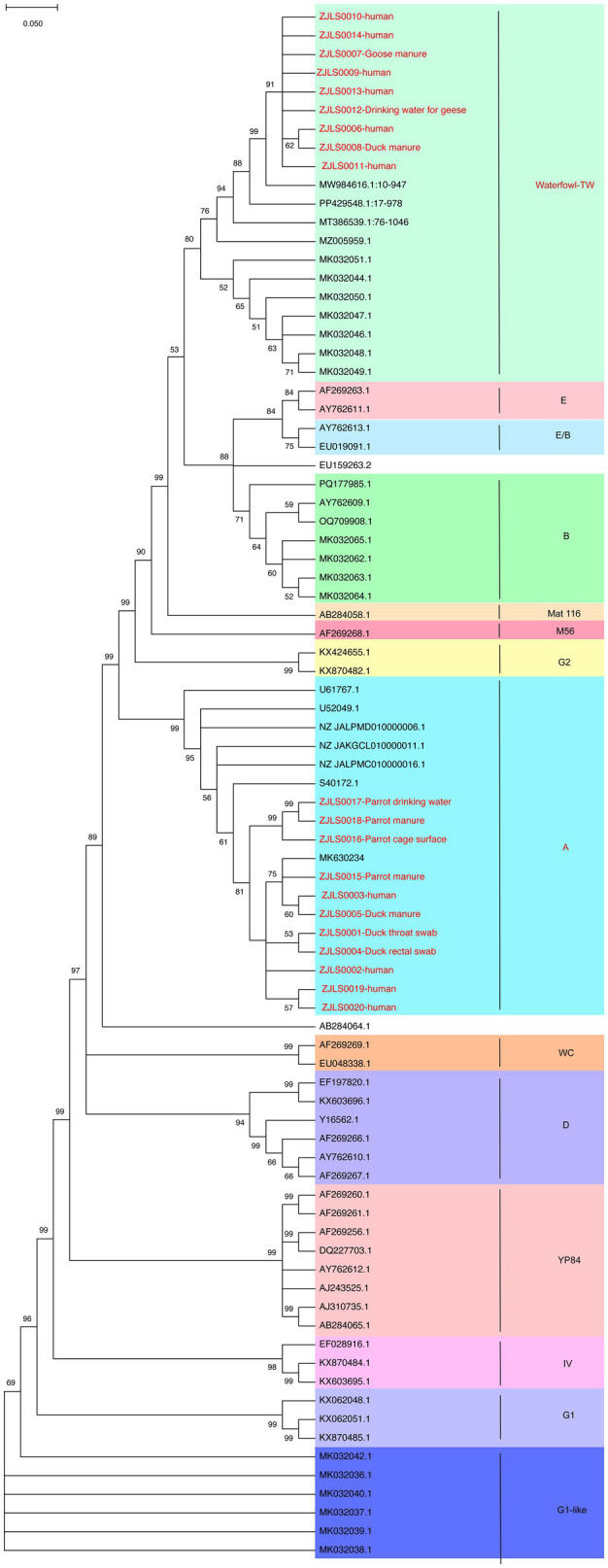
Phylogenetic tree based on *ompA* gene sequences of 20 *C. psittaci* isolates and 64 reference strains. The analysis resolved the isolates into two clades: eleven sequences clustered with MK630234 (genotype A), while nine sequences grouped with MW984616.1 (genotype waterfowl-TW). Bootstrap support values (1,000 replicates) are shown at major nodes. Values ≥70% indicate strong support.

A cluster was defined as a minimum of two epidemiologically linked cases within a specified period, which resulted in expanded field investigations. A comprehensive set of clinical, household, animal, and environmental samples was collected, and exposure timelines were meticulously constructed to elucidate the transmission pathways. For phylogenetic analysis, 20 ompA gene sequences (ZJLS001–ZJLS020) were selected for amplification from 28 *C. psittaci*-positive samples via nested PCR. The 20 ompA sequences (ZJLS001–ZJLS020) have been deposited in National Microbiology Data Center under accession numbers NMDC10020636. This selection was based on the quality and coverage criteria of the sequences. Sequences with >60% coverage and an average sequencing depth greater than 1 were aligned against 64 ompA reference sequences from NCBI GenBank using MUSCLE in MEGA v12. Phylogenetic trees were constructed utilizing the neighbor-joining method and the Tajima–Nei model, with bootstrap analysis (1,000 replicates) was performed, and bootstrap support values ≥70% are displayed at corresponding nodes in Figure 1.

### Quality control and statistical methods

In order to reduce misclassification, exclusively laboratory-confirmed cases were included in the study. The process of data abstraction was conducted in accordance with a standardized protocol, which involved dual review of key dates. The administration of structured questionnaires served to minimize variability in the ascertainment of exposure among interviewers. The issue of environmental under-ascertainment was mitigated by the analysis of multiple sample types per site. Sensitivity analyses were conducted to evaluate the robustness of summary intervals, with mean and median values being utilized for this purpose. A compendium of metrics was collated for the purpose of summarizing demographics, clinical features, exposures, and time intervals. Categorical variables were reported as counts and percentages; A structured database was established using Microsoft Excel 2019, and statistical analyses were performed with SPSS version 23.0 (IBM Corp., Armonk, NY, United States). Descriptive analyses were conducted to summarize the temporal, geographic, and demographic distribution of *psittacosis* cases, as well as clinical characteristics and diagnostic intervals. Continuous variables were assessed for normality and presented as means ± standard deviation (SD) when normally distributed, or as medians with range or interquartile range (IQR) when non-normally distributed. Categorical variables were expressed as frequencies and percentages. Comparisons of proportions were performed using the χ^2^ test or Fisher’s exact test where appropriate. Non-parametric comparisons between two groups were conducted using the Mann–Whitney U test. A two-sided significance level of α = 0.05 was applied. Given the relatively small sample size, analyses were primarily descriptive, and inferential statistical testing was interpreted cautiously. The degree of environmental positivity was determined as the proportion of positive samples in the overall PCR results. Temporal trends were described on a monthly and annual basis, while geographic distributions were aggregated at the county level.

## Results

### Epidemiological overview of *psittacosis* cases

**Figure 2:**
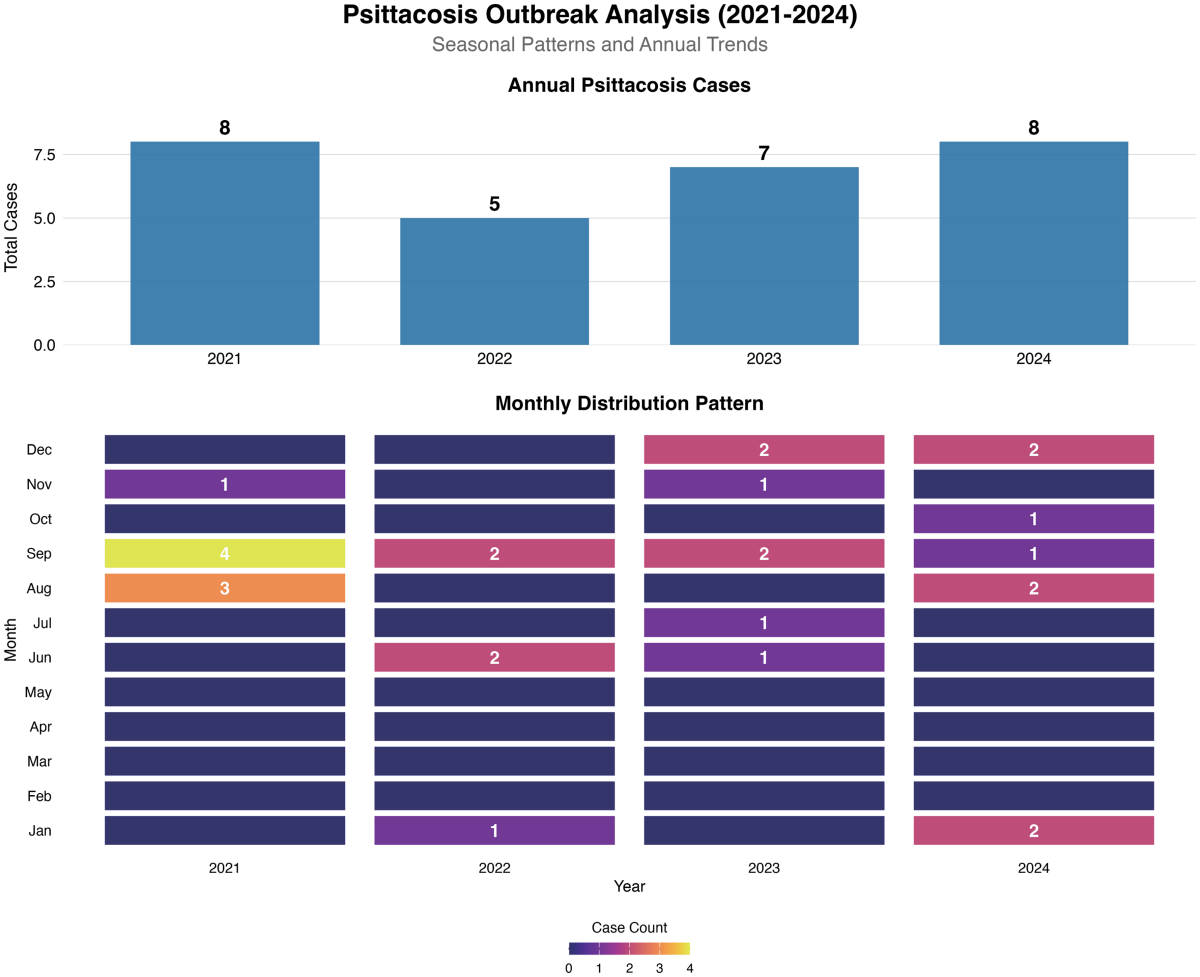
Statistics of *psittacosis* cases in Lishui City, Zhejiang Province, China, during the period August 2021–December 2024. The highest monthly case counts were observed in August (3 cases) and September (4 cases) 2021, representing the numerical monthly peak during the study period, respectively, and the rest of the months had 2 cases or fewer.

From August 2021 to December 2024, a total of 28 laboratory-confirmed human *psittacosis* cases were reported in Lishui City, Zhejiang Province. The annual distribution of cases was as follows: 8 cases in 2021 (all occurring between August and December), 5 cases in 2022, 7 cases in 2023, and 8 cases in 2024. Notably, a peak in the number of cases was observed between August and September 2021 (Figure 2).

### Geographical distribution of patients

**Figure 3:**
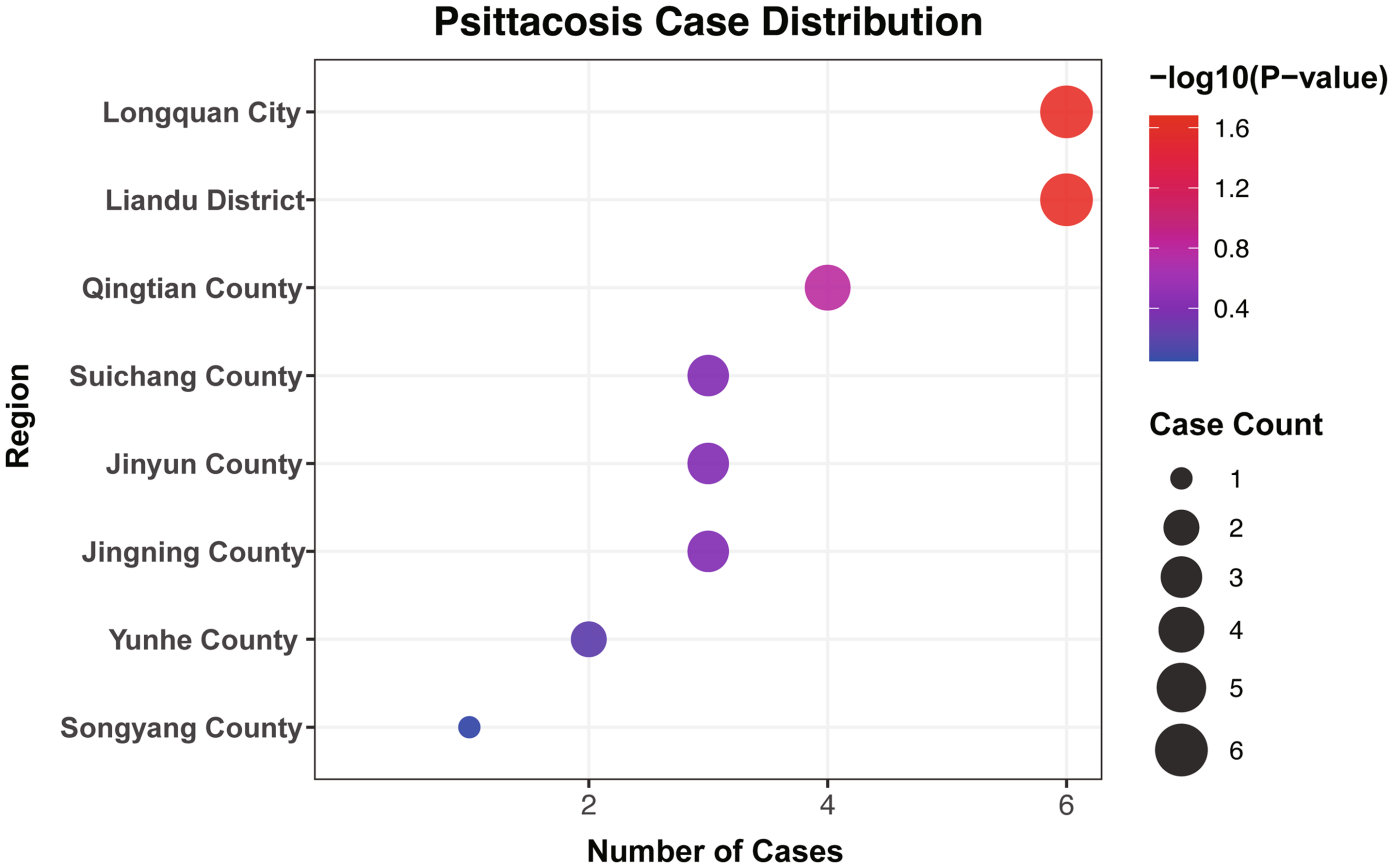
The incidence of *psittacosis* in various districts and counties.

During the study period, cases were geographically dispersed, with reports originating from eight of the nine counties (excluding Qingyuan). Liandu District and Longquan City each accounted for six cases, while Qintian, Jinyun, Jingning, and Suichang accounted for four, three, three, and three cases, respectively; Yunhe and Songyang accounted for two and one case, respectively (Figure 3). Figure 3 presents a descriptive geographic distribution and does not imply formal spatial clustering analysis. Overall, 15.03% (26/173) of the townships (subdistricts) within the jurisdiction had reported cases. Specifically, these cases were distributed across 26 villages located within those 26 townships (subdistricts). One village reported 3 cases, while each of the other 25 villages reported only 1 case, indicating a highly sporadic distribution (Supplementary Figure S1).

### Epidemiological characteristics of *psittacosis* cases

The median age of patients was 62.5 years (range 40–77; mean 62.6 ± 9.7), with males comprising 53.6% of the cohort. The age-specific incidence rates of *psittacosis* cases are presented in Supplementary Figure S2. Farmers accounted for 75.0% (21/28) of cases, with 27/28 (96.4%) reporting recent contact with poultry. Detailed exposure histories were available for 27 of the 28 cases (96.4%), all of whom reported contact with birds or poultry within 30 days prior to symptom onset. The most common exposure was backyard poultry breeding, including raising chickens and ducks (*n* = 17), chickens only (*n* = 3), ducks only (*n* = 1), and combined exposure to chickens and wild birds (*n* = 1). Additional exposures included raising parrots (*n* = 1) and pigeons (*n* = 1). High-risk activities such as slaughtering poultry (*n* = 1) and cleaning poultry enclosures (*n* = 1) were also reported. One case developed symptoms after providing care for an infected household member. Exposure history was unclear in one case. The median interval from symptom onset to the first medical consultation was 3 days (IQR 4.5). From initial consultation to laboratory confirmation, the median delay was 8.5 days (IQR 7.25). This resulted in a median total time from symptom onset to diagnosis was 12 days (IQR 9.25). Patients were hospitalized for a median of 17 days (IQR 9.75) (Table 1).

**Table 1:** Patient population and epidemiological characteristics.

Variables	*N* = 28
Sex [*N* (%)]	
Male	15 (53.57)
Female	13 (46.43)
Gender ratio of men to women	1:0.8
Median age (years)	62.50 (15.25)
Median number of episodes to consultations (days)	3 (4.5)
Median consultation to diagnosis (days)	8.5 (7.25)
Median time from case onset to diagnosis (days)	12 (7.25)
Median hospitalization (days)	17 (9.75)
Median disease duration (days)	22.5 (11.5)
Occupation [*N* (%)]	
Farmer	21 (75.00)
Individual business	3 (10.71)
Worker	2 (7.14)
Retired personnel	1 (3.57)
Company employee	1 (3.57)
Exposure history [*N* (%)]	
Contact with poultry	27 (96.43)
Not clear	1 (3.57)

### Clinical manifestations

All patients presented with fever (28/28); other common symptoms included cough (22/28, 78.57%), chills (20/28, 71.43%), shortness of breath (18/28, 64.29%), and fatigue (16/28, 57.14%). Eighteen patients (64.29%) were diagnosed with severe pneumonia (Table 2). A comparative analysis between severe and non-severe cases was conducted using the Mann–Whitney U test. No statistically significant difference was observed in the diagnostic interval between the two groups (*Z* = −1.370, *p* = 0.171). However, age differed significantly between severe and non-severe patients (*Z* = −2.064, *p* = 0.039), with patients in the severe group being older.

**Table 2:** Clinical manifestations of patients [*N* (%)].

Variables	*N* = 28
Fever	28 (100)
Cough	22 (78.57)
Chills	20 (71.43)
Shortness of breath	18 (64.29)
Fatigue	16 (57.14)
Headache	7 (25.00)
Myalgia	6 (21.43)
Severe pneumonia	18 (64.29)

### Monitoring of close contacts, poultry and environmental samples

**Figure 4:**
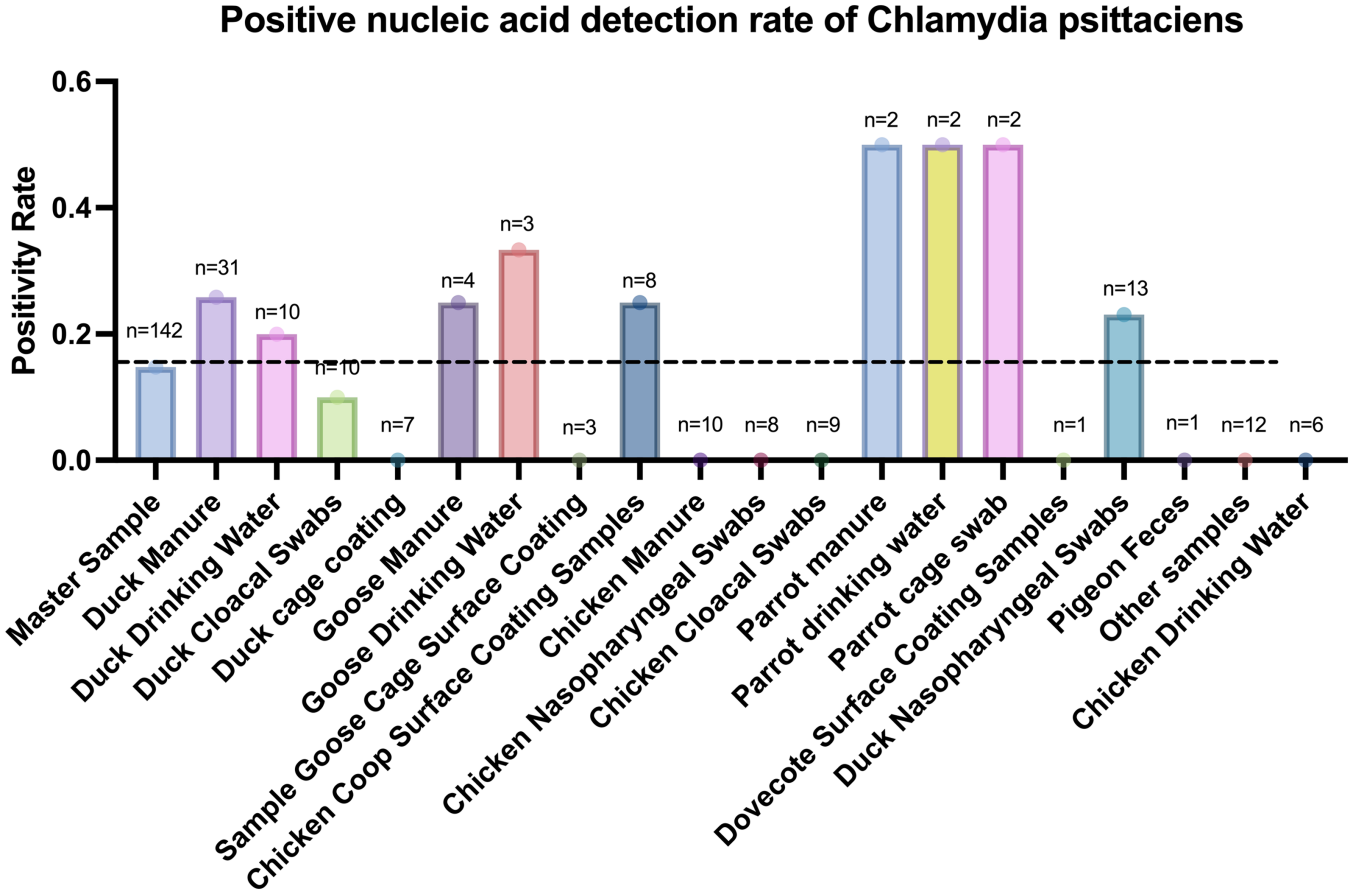
Detection rate of *psittacosis* in environmental samples.

A total of 205 close contacts were placed under 14-day medical observation, and none developed clinical symptoms during the monitoring period. Concurrently, environmental and avian sampling was conducted, including 142 specimens (nasopharyngeal and cloacal swabs, feces, drinking water, and cage equipment). Among these specimens, 21 samples (14.79%) tested positive for *C. psittaci* nucleic acid, with duck feces showing the higher positivity rate (26.7%). Further details are provided in Figure 4. A detailed summary of confirmed cases and their corresponding environmental sampling information, reorganized by exposure events and epidemiological clusters to illustrate human-environment linkages, is provided in Supplementary Table S2.

### Identification and characteristics of a case cluster

One familial cluster comprising three related individuals was identified in Dazhangshu Village, Chatian Town, Longquan City, in September 2021. Two cases had direct exposure to domestic poultry, including household chickens and ducks, while the third case adult son who reported no occupational exposure to poultry, developed symptoms after providing care for his ill parents. During the investigation, 14 samples were collected from poultry and household environments, of which six (42.86%) were *C. psittaci*–positive. The details of this cluster outbreak case can be found in our previous article (A cluster of *Psittacosis* cases in Lishui, Zhejiang Province, China, in 2021).

### Phylogenetic analysis and genotyping

To determine the genotypes of the 20 *C. psittaci* PCR-positive samples, the amplified ompA gene products were sequenced and subjected to BLAST analysis against reference sequences. A phylogenetic tree was constructed using these 20 sequences with 64 reference sequences (Figure 1). The phylogenetic analysis demonstrated that the 20 sequences were resolved into two distinct clades. Specifically, the first 11 sequences were grouped within a single cluster, showing a close evolutionary relationship with the MK630234 sequence, and were highly homologous to *C. psittaci* genotype A. By contrast, the remaining nine sequences were positioned within a separate clade, exhibited the closest affinity with the MW984616.1 sequence, and were identified as belonging to *C. psittaci* genotype waterfowl-TW.

## Discussion

*Psittacosis* is a significant zoonotic disease that presents sporadically in China, particularly among individuals frequently exposed to domestic poultry (Liu et al., 2023). The annual distribution of cases during the study period reflects fluctuation rather than a consistent upward trajectory. In the present investigation, we identified 28 laboratory-confirmed cases of *Chlamydia psittaci* in Lishui City, Zhejiang Province, between August 2021 and 2024; of these, 20 ompA-positive specimens were successfully sequenced for genotyping. Given the rural, mountainous context characterized by widespread backyard poultry rearing and limited diagnostic resources, the findings should be interpreted as region-specific and may not be readily generalizable beyond comparable settings in southeastern China (Yan et al., 2018). Our epidemiological analysis revealed that the majority of cases occurred among middle-aged and elderly farmers, with a seasonal concentration in late summer and early autumn. These observations suggest that occupational exposure and backyard poultry husbandry represent the predominant risk factors in this region, which is broadly consistent with trends documented in both domestic and international reports (Ossa-Giraldo et al., 2023; Rybarczyk et al., 2020; Vorimore et al., 2015). Similar demographic patterns have been reported in recent studies from Zhejiang Province and other regions of China, where elderly farmers with frequent exposure to backyard poultry constituted the primary affected population (Ding et al., 2025; Wang et al., 2025). These findings suggest that occupational and household contact with domestic birds remains a major risk factor for human *psittacosis* in rural settings (Liu et al., 2023).

The phylogenetic analysis of ompA gene sequences further indicated that the clinical isolates clustered within genotypes A and waterfowl-TW, both of which exhibited close genetic similarity to reference strains previously described in southern China (Liu et al., 2019; Lu et al., 2023). Genotype A has historically been associated with psittacine birds and has frequently been detected in human infections, whereas waterfowl-associated genotypes have increasingly been identified in domestic ducks and geese across East Asia (Hogerwerf et al., 2020; Zhang et al., 2015). These host-associated genotype patterns suggest that multiple avian reservoirs may contribute to human exposure in rural environments (Long et al., 2025; Zhang et al., 2024). Specifically, 11 sequences clustered with MK630234 (genotype A), whereas nine showed the highest similarity to MW984616.1 (genotype waterfowl-TW). These findings suggest that ducks and geese may represent important reservoirs and plausible sources of human infection; however, direct transmission pathways cannot be conclusively established based solely on phylogenetic similarity. Previous investigations have demonstrated relatively high detection rates of *C. psittaci* in waterfowl populations, particularly in domestic ducks and geese, supporting their potential role as important reservoirs for zoonotic transmission (Long et al., 2025; Zhao et al., 2015). Backyard poultry farming practices may further facilitate environmental contamination and human exposure (Dickx et al., 2013). Of particular note, the identification of a familial cluster, in which one patient lacked direct poultry exposure, raises the possibility of limited human-to-human transmission; however, this remains unproven. Importantly, no symptomatic secondary infections were identified among 205 monitored close contacts. Nevertheless, this interpretation should be treated with caution, as such events are rarely reported, and our own monitoring of 205 close contacts revealed no symptomatic secondary infections. However, the absence of clinically apparent illness does not exclude the possibility of asymptomatic or subclinical infection, as systematic PCR or serological testing was not performed among close contacts.

From a clinical perspective, *psittacosis* in this cohort presented with non-specific manifestations, most frequently fever, cough, and dyspnea, thereby contributing to frequent misdiagnosis as influenza or community-acquired pneumonia (Beeckman and Vanrompay, 2009; Li et al., 2022; Wang et al., 2024). More than half of the patients progressed to severe pneumonia, and three fatalities were recorded among elderly or immunocompromised individuals. In our cohort, older age was significantly associated with severe disease, as patients in the severe group were older than those in the non-severe group (Liu et al., 2025; Tian et al., 2025). These outcomes are in line with previous reports of substantial morbidity and mortality associated with delayed recognition of *psittacosis* (de Perio et al., 2020; Zhang et al., 2022). Delayed recognition and inappropriate empirical antibiotic therapy have been identified as major contributors to disease progression and severe pneumonia in *psittacosis* (Shi et al., 2021; Stewardson and Grayson, 2010). Because clinical manifestations are nonspecific, the disease is frequently misdiagnosed as other forms of community-acquired pneumonia, resulting in delayed initiation of effective tetracycline therapy (He et al., 2023; Li et al., 2021). The median diagnostic delay of more than 10 days in our study underscores the pressing need to enhance clinician awareness. While metagenomic next-generation sequencing was critical for case confirmation (Tang et al., 2022), its high cost and limited accessibility restrict routine use. Broader adoption of targeted molecular assays, such as qPCR, may therefore represent a more feasible strategy for timely case identification in local healthcare facilities.

At the international level, an unusual increase in *psittacosis* notifications was reported in late 2023–early 2024 across several European countries, including Austria, Denmark, Germany, Sweden, and the Netherlands. Five deaths were documented, with most patients reporting exposure to wild and/or domestic birds. These observations most likely reflect seasonal factors, levels of avian exposure, and increased diagnostic awareness. Our findings from Lishui show parallels in terms of bird-associated risk and seasonal concentration, although the host ecology differs, with ducks and other domestic waterfowl serving as the principal reservoirs (environmental positivity: 14.79% overall; 26.7% in duck feces). In contrast, surveillance data from the United States indicate low but steady annual case numbers without a continent-wide surge comparable to that observed in Europe. In Asia, a cluster of human-to-human transmissions (China, 2020; published 2022) highlights that person-to-person spread can occur in situations of high exposure, although our active monitoring of 205 close contacts revealed no secondary cases with symptoms. Taken together, these comparisons suggest that avian ecology, exposure practices, and diagnostic system capacity jointly shape the epidemiology of *psittacosis* across different regions.

This study has several important public health implications. First, community-level education programs directed at rural farmers and poultry handlers should be strengthened, particularly to promote protective behaviors and to minimize direct contact with sick or dead birds. Second, environmental surveillance targeting domestic waterfowl populations and their habitats should be reinforced, given the relatively high detection rates in duck feces and drinking water observed in this study. Third, clinicians should be encouraged to consider *psittacosis* in the differential diagnosis of febrile respiratory illness during seasonal peaks and to promptly initiate tetracycline-class therapy when clinically suspected, in alignment with local guidelines and antimicrobial stewardship principles. Although this study incorporated human case investigation with environmental and poultry sampling, it was primarily human-case–triggered rather than based on simultaneous, systematic human–animal–environment surveillance. Future studies should implement longitudinal, integrated sampling across human, domestic animal, wild bird, and environmental interfaces to fully operationalize a One Health surveillance framework (Tegegne et al., 2023).

Several limitations of this study should be acknowledged. First, because this investigation relied on passive laboratory-confirmed case reporting, the true incidence of *psittacosis* is likely underestimated, particularly in rural areas with limited access to molecular diagnostics. Observed annual fluctuations in reported case numbers may partially reflect enhanced clinical awareness and expanded use of mNGS rather than true changes in transmission intensity. In addition, because only laboratory-confirmed cases were included, milder or undiagnosed infections may have been missed, potentially leading to overestimation of the proportion of severe disease. Consequently, incidence patterns and risk factor analyses should be interpreted with caution. Second, environmental sampling did not fully encompass all potential exposure pathways and was conducted as case-triggered convenience sampling, which limits generalizability. Moreover, PCR and mNGS detect nucleic acid but do not confirm bacterial viability; therefore, environmental positivity should be interpreted as evidence of contamination rather than confirmed active transmission. Third, although phylogenetic analysis demonstrated similarity between human and poultry-derived *C. psittaci* sequences, such genetic relatedness does not establish direct zoonotic transmission chains. Finally, due to the relatively small sample size, inferential statistical analyses for robust risk factor identification were not performed, and the exploratory analyses presented should be interpreted cautiously. Future studies with larger sample sizes are warranted to validate these findings and provide more definitive evidence. Metagenomic next-generation sequencing has increasingly been applied in the diagnosis of atypical pathogens responsible for community-acquired pneumonia (Li et al., 2021; Shi et al., 2024). Several studies have demonstrated that mNGS significantly improves the detection of *C. psittaci* compared with conventional diagnostic methods, particularly in severe or atypical cases where routine testing fails to identify the causative pathogen (Wang et al., 2022).

In conclusion, *psittacosis* in Lishui City is characterized by sporadic but clinically significant infections primarily associated with backyard poultry exposure. Elderly rural residents appear to constitute the most vulnerable population, and diagnostic delays continue to adversely influence clinical outcomes. These findings highlight the urgent need to strengthen clinical recognition, expand diagnostic capacity, and reinforce environmental monitoring. The evidence presented here is region-specific and may inform prevention and control strategies in Lishui and other comparable settings with backyard poultry husbandry. Extrapolation to other regions should be undertaken with caution and adapted to local ecological conditions, diagnostic infrastructure, and health system capacity.

## Data Availability

The datasets presented in this study can be found in online repositories. The names of the repository/repositories and accession number(s) can be found in the article/Supplementary material.
