# Supplementary material for: Epidemiological characteristics and environmental surveillance of human psittacosis in Lishui City, Zhejiang Province, China (2021–2024)
Source: Front Microbiol. 2026 Apr 8;17:1769696. doi: 10.3389/fmicb.2026.1769696 (PMC13099532; doi:10.3389/fmicb.2026.1769696)
Supplement: Supplementary file 1 [file Supplementary_file_1.docx]

**Epidemiological characteristics and environmental surveillance of**

**human psittacosis in Lishui City, Zhejiang Province, China(2021–2024)**

**Supplementary material**

**Supplementary Fig.1** The distribution of parrot fever in townships.

**
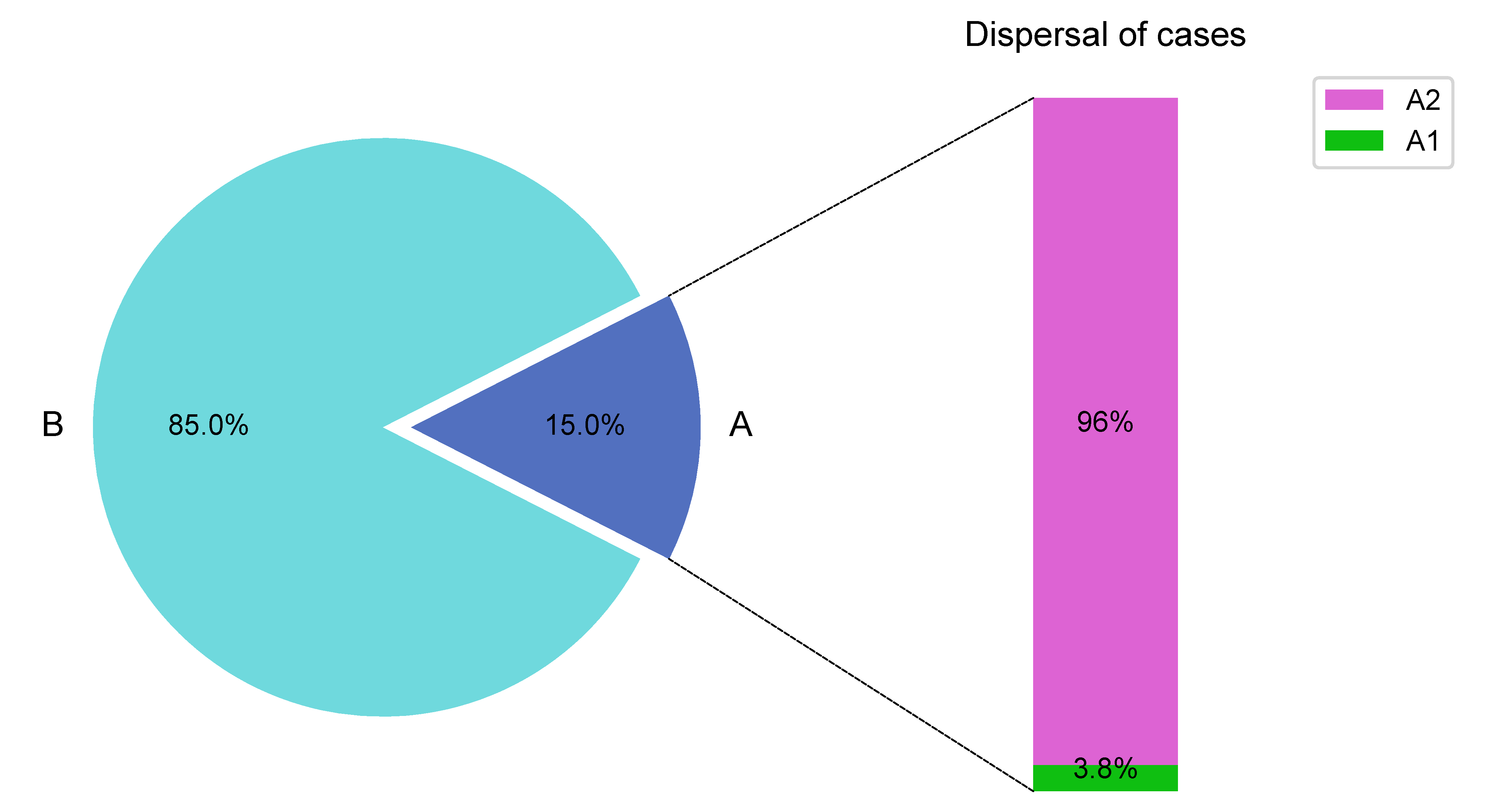
**

A represents the proportion of townships with parrot fever cases in the total number of townships; A1 represents the proportion of three cases in the same township in the townships with cases; A2 represents the proportion of one case in the same township in the townships with cases.

**Supplementary Fig.2** Incidence rates by age group.

**
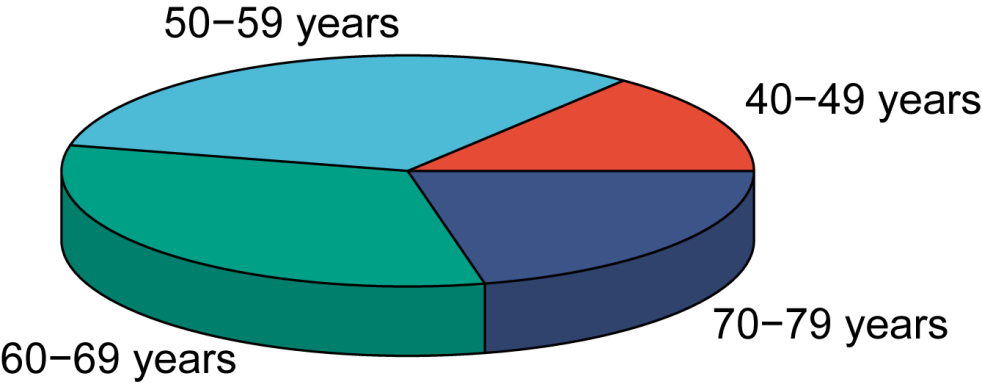
**

The age groups with the highest incidence were the 50–59 and 60–69 cohorts, with nine cases in each. The 70–79 age group had 6 cases, while the 40–49 age group had 4. No cases were reported among individuals under 40.

**Supplementary Table 1: The primers and probes used are displayed.**

| Primer name | Sequence 5’-3’ | Amplicon size(bp) |
| --- | --- | --- |
| Nest-1-F | GCTACGGGTTCCGCTCT | 1017 |
| Nest-1-R | GCTTCGATTCAGATCAACAAA |  |
| Nest-2-F | CGCTCTCTCCTTACAAGCC | 1000 |
| Nest-2-R | CAAATTGCTTCGATTCAGATC |  |
| Realtime-F | CACTATGTGGGAAGGTGCTTCA | 76 |
| Realtime-R | CTGCGCGGATGCTAATGG |  |
| Realtime-pb | FAM- CGCTACTTGGTGTGAC-BHQ1 |  |

**Supplementary Table 2:** A detailed summary of confirmed cases and their corresponding environmental sampling information.

|  | **Date of onset** | **Date of diagnosis** | **Detection Method** | **result** |
| --- | --- | --- | --- | --- |
| Patient 1 | September 3, 2021 | September 13, 2021 | Metagenomic Sequencing | positive |
| Patient 2 | September 4, 2021 | September 13, 2021 | Metagenomic Sequencing | positive |
| Patient 3 | September 13, 2021 | September 29, 2021 | Metagenomic Sequencing | positive |
| Patient 4 | August 18, 2021 | September 9, 2021 | Metagenomic Sequencing | positive |
| Patient 5 | August 18, 2021 | September 9, 2021 | Metagenomic Sequencing | positive |
| Patient 6 | August 28, 2021 | September 10, 2021 | Metagenomic Sequencing | positive |
| Patient 7 | September 5, 2021 | September 23, 2021 | Metagenomic Sequencing | positive |
| Patient 8 | November 28, 2021 | December 5, 2021 | Metagenomic Sequencing | positive |
| Patient 9 | January 13, 2022 | January 21, 2022 | Metagenomic Sequencing | positive |
| Patient 10 | June 15, 2022 | June 29, 2022 | Metagenomic Sequencing | positive |
| Patient 11 | June 26, 2022 | July 12, 2022 | Metagenomic Sequencing | positive |
| Patient 12 | September 3, 2022 | September 21, 2022 | Metagenomic Sequencing | positive |
| Patient 13 | September 15, 2022 | September 23, 2022 | Metagenomic Sequencing | positive |
| Patient 14 | July 12, 2023 | July 20, 2023 | Metagenomic Sequencing | positive |
| Patient 15 | June 22, 2023 | July 20, 2023 | Metagenomic Sequencing | positive |
| Patient 16 | September 8, 2023 | September 19, 2023 | Metagenomic Sequencing | positive |
| Patient 17 | September 28, 2023 | October 9, 2023 | Metagenomic Sequencing | positive |
| Patient 18 | November 11, 2023 | December 4, 2023 | Metagenomic Sequencing | positive |
| Patient 19 | December 5, 2023 | December 8, 2023 | Metagenomic Sequencing | positive |
| Patient 20 | December 25, 2023 | January 16, 2023 | Metagenomic Sequencing | positive |
| Patient 21 | January 19, 2024 | January 30, 2024 | Metagenomic Sequencing | positive |
| Patient 22 | January 17, 2024 | January 31, 2024 | Metagenomic Sequencing | positive |
| Patient 23 | August 13, 2024 | August 22, 2024 | Metagenomic Sequencing | positive |
| Patient 24 | August 7, 2024 | August 14, 2024 | Metagenomic Sequencing | positive |
| Patient 25 | September 15, 2024 | September 30, 2024 | Metagenomic Sequencing | positive |
| Patient 26 | October 9, 2024 | October 17, 2024 | Metagenomic Sequencing | positive |
| Patient 27 | December 4, 2024 | December 13, 2024 | Metagenomic Sequencing | positive |
| Patient 28 | December 4, 2024 | December 24, 2024 | Metagenomic Sequencing | positive |
| Duck drinking water | September 17, 2021 | September 24, 2021 | Nested PCR | positive |
| Duck manure | September 17, 2021 | September 24, 2021 | Nested PCR | positive |
| Goose Manure | September 17, 2021 | September 24, 2021 | Nested PCR | positive |
| Goose Drinking Water | September 17, 2021 | September 24, 2021 | Nested PCR | positive |
| Duck Nasopharyngeal Swabs | September 23, 2022 | September 23, 2022 | Nested PCR | positive |
| Duck Cloacal Swabs | September 29, 2022 | September 29, 2022 | Nested PCR | positive |
| Duck cloacal swab | June 30, 2022 | June 30, 2022 | Nested PCR | positive |
| Duck nasal and oropharyngeal swabs | June 30, 2022 | June 30, 2022 | Nested PCR | positive |
| Parrot mannrue | September 23, 2024 | September 23, 2024 | Nested PCR | positive |
| Parrot drinking water | September 23, 2024 | September 23, 2024 | Nested PCR | positive |
| Parrot cage swab | September 23, 2024 | September 23, 2024 | Nested PCR | positive |

| case | Sampling time | Sampling location | Specimen type | Detection quantity | Positive count |
| --- | --- | --- | --- | --- | --- |
| case1、case2 | September 10, 2021 | The Sick Family in Dazhangshu Village, Chatian Town, Longquan City | Duck manure | 3 | 2 |
|  |  |  | Duck nasal and oropharyngeal swabs | 6 | 3 |
|  |  |  | Duck drinking water | 1 | 1 |
|  |  |  | Other Samples | 4 | 0 |
|  |  |  | Total | 14 | 6 |
| case3 | September 10, 2021 | The Sick Family in Banguyue Village, Chishou Township, Songyang County | Duck manure | 4 | 0 |
|  |  |  | Duck drinking water | 3 | 0 |
|  |  |  | Duck cage swab | 3 | 0 |
|  |  |  | Goose Manure | 4 | 1 |
|  |  |  | Goose Drinking Water | 3 | 1 |
|  |  |  | Goose cage swab | 3 | 0 |
|  |  |  | Chicken manure | 4 | 0 |
|  |  |  | Chicken drinking water | 3 | 0 |
|  |  |  | Chicken coop swab | 3 | 0 |
|  |  |  | Total | 30 | 2 |
| case4 | September 28, 2021 | Kengding Village, Yuanhe Subdistrict, Yunhe County | Duck manure | 3 | 1 |
|  |  |  | Duck cage swab | 4 | 0 |
|  |  |  | Duck drinking water | 2 | 0 |
|  |  |  | Chicken manure | 3 | 0 |
|  |  |  | Chicken drinking water | 2 | 0 |
|  |  |  | Chicken coop swab | 2 | 2 |
|  |  |  | Other Samples | 2 | 0 |
|  |  |  | Total | 18 | 3 |
| case5 | September 29, 2021 | Tongtou Village, Lanjù Township, Longquan City | Chicken cloacal swab | 9 | 0 |
|  |  |  | Chicken coop swab | 3 | 0 |
|  |  |  | Chicken nose, oropharyngeal swab | 3 | 0 |
|  |  |  | Chicken drinking water | 1 | 0 |
|  |  |  | Chicken manure | 1 | 0 |
|  |  |  | Other Samples | 4 | 0 |
|  |  |  | Total | 21 | 0 |
| case1、case2 | September 29, 2021 | The Other villagers in Dazhangshu Village, Chatian Town, Longquan City | Duck manure | 21 | 5 |
|  |  |  | Duck nasal and oropharyngeal swabs | 4 | 0 |
|  |  |  | Duck cloacal swab | 2 | 1 |
|  |  |  | Duck drinking water | 1 | 1 |
|  |  |  | Other Samples | 2 | 0 |
|  |  |  | Total | 30 | 7 |
| case6 | December 6, 2021 | The Sick Family in Dadi Village, Dadi Township, Jingning County | pigeon droppings | 1 | 0 |
|  |  |  | Pigeon Cage Swab | 1 | 0 |
|  |  |  | Total | 2 | 0 |
| case7 | June 30, 2022 | The Sick Family in Gaosha Village, Haikou Town, Qingtian County | Duck cloacal swab | 8 | 0 |
|  |  |  | Duck nasal and oropharyngeal swabs | 3 | 0 |
|  |  |  | Duck drinking water | 3 | 0 |
|  |  |  | Chicken nose, oropharyngeal swab | 5 | 0 |
|  |  |  | Chicken manure | 2 | 0 |
|  |  |  | Total | 21 | 0 |
| case8 | September 23, 2024 | Lian'du District, Wanxiang Subdistrict, Dengtashan Street, Patient's Residence | Parrot manure | 2 | 1 |
|  |  |  | Parrot drinking water | 2 | 1 |
|  |  |  | Parrot cage swab | 2 | 1 |
|  |  |  | Total | 6 | 3 |

| Year | 2021 | 2022 | 2023 | 2024 |
| --- | --- | --- | --- | --- |
| Number of Cases | 5 | 5 | 7 | 8 |

| Region | Jinyun County | Jingning County | Liandu District | Qingtian County | Suichang County | Songyang County | Longquan City | Yunhe County |
| --- | --- | --- | --- | --- | --- | --- | --- | --- |
| Number of cases | 3 | 3 | 6 | 4 | 2 | 1 | 4 | 2 |

| sampling time | sampling position | Number of tests | Number of positive cases | Detection rate(%) |
| --- | --- | --- | --- | --- |
| September 10, 2021 | The Sick Family in Dazhangshu Village, Chatian Town, Longquan City | 14 | 6 | 42.86 |
| September 10, 2021 | The Sick Family in Banguyue Village, Chishou Township, Songyang County | 30 | 2 | 6.67 |
| September 28, 2021 | Kengding Village, Yuanhe Subdistrict, Yunhe County | 18 | 3 | 16.67 |
| September 29, 2021 | Tongtou Village, Lanjù Township, Longquan City | 21 | 0 | 0 |
| September 29, 2021 | Other villagers of Dazhangshu Village, Chatian Town, Longquan City | 30 | 7 | 23.33 |
| December 7, 2021 | The Sick Family in Dadi Village, Dadi Township, Jingning County | 2 | 0 | 0 |
| June 30, 2022 | The Sick Family in Gaosha Village, Haikou Town, Qingtian County | 21 | 0 | 0 |
| September 23, 2024 | Lian'du District, Wanxiang Subdistrict, Dengtashan Street, Patient's Residence | 6 | 3 | 50 |
| Total |  | 142 | 21 | 14.79 |
